# Interferon-Induced Transmembrane Protein 1 (IFITM1) Is Downregulated in Neurofibromatosis Type 1-Associated Malignant Peripheral Nerve Sheath Tumors

**DOI:** 10.3390/ijms25179265

**Published:** 2024-08-27

**Authors:** Gun-Hoo Park, Eunkuk Park, Su-Jin Lee, Kyubin Lim, Jeonghyun Kim, Jun Eun Park, Seon-Yong Jeong

**Affiliations:** 1 Department of Medical Genetics, Ajou University School of Medicine, Suwon 16499, Republic of Korea; gunhoopark@kiost.ac.kr (G.-H.P.); jude0815@ajou.ac.kr (E.P.); sujinlee5699@gmail.com (S.-J.L.);; 2 Jeju Bio Research Center, Jeju Research Institute, Korea Institute of Ocean Science & Technology (KIOST), Jeju-si 63349, Republic of Korea; 3 Jeonbuk Institute for Food-Bioindustry, Jeonju 54810, Republic of Korea; 4 Department of Biomedical Sciences, Ajou University Graduate School of Medicine, Suwon 16499, Republic of Korea; 5 Department of Pediatrics, Anam Hospital, Korea University College of Medicine, Seoul 02841, Republic of Korea

**Keywords:** neurofibromatosis type 1 (NF1), malignant peripheral nerve sheet tumor (MPNST), tumor progression, interferon-induced transmembrane protein 1 (IFITM1), interferon-gamma (IFN-γ)

## Abstract

Neurofibromatosis type 1 (NF1), an autosomal dominant genetic disorder, is caused by mutations in the *NF1* gene, which encodes the GTPase-activating protein neurofibromin. The pathogenesis of the tumor progression of benign plexiform neurofibromas (PNs) and malignant peripheral nerve sheath tumors (MPNSTs) remain unclear. Here, we found that interferon-induced transmembrane protein 1 (IFITM1) was downregulated in MPNST tissues compared to those in PN tissues from patients with NF1. Overexpression of IFITM1 in NF1-associated MPNST cells resulted in a significant decrease in Ras activation (GTP-Ras) and downstream extracellular regulatory kinase 1/2 (ERK1/2) phosphorylation, whereas downregulation of IFITM1 via treatment with small interfering RNA in normal Schwann cells had the opposite result, indicating that expression levels of IFITM1 are closely associated with tumor progression in NF1. Treatment of MPNST cells with interferon-gamma (IFN-γ) significantly augmented the expression of IFITM1, thereby leading to a decrease in Ras and ERK1/2 activation. Despite the small number of patient samples, these findings may potentially provide a new target for chemotherapy in patients with NF1-associated MPNSTs. In xenograft mice injected with MPNST cells, *IFN-*γ treatment successfully suppressed tumor progression with increased IFITM1 expression and decreased Ras and ERK1/2 activation in tumor tissues. Collectively, these results suggest that IFITM1 is closely involved in MPNST pathogenesis and that IFN-γ is a good candidate for the therapeutic treatment of MPNSTs in NF1.

## 1. Introduction

Neurofibromatosis type 1 (NF1), also known as von Recklinghausen disease, is an autosomal dominant genetic disorder that affects 1 in approximately 3500 individuals worldwide [1,2]. Common clinical manifestations of NF1 include Lisch nodules, bone dysplasia, café-au-lait spots, axillary freckling, optic nerve glioma, and nerve sheath tumors [3]. NF1 is caused by a mutation in *NF1*, which encodes the Ras-GTPase tumor suppressor neurofibromin [4]. Malignant peripheral nerve sheath tumor (MPNST) is a highly invasive sarcoma of soft tissues, with a 5-year overall survival rate of 15–66% in patients with NF1; however, surgical resection is the only treatment that can increase the survival rate [5,6]. The possibility of patients with NF1 developing MPNST has been estimated to be 8–13% [7] and 5.9–10.3% [8]. MPNSTs are strongly associated with internal plexiform neurofibromas (PNs) [9], indicating that tumor progression is a major concern in patients with NF1 [10]. Previous studies suggested that loss of heterozygosity (LOH) of the *NF1* gene in Schwann cells is a key factor in developing neurofibromas [11,12]. However, *NF1* locus LOH in Schwann cells is frequently observed in benign plexiform neurofibromas [13], suggesting that additional modifier factors may be involved in the malignant transformation of benign tumors to MPNST.

Many studies noted that genetic mutations or changes in the expression of tumor protein of key genes are commonly found in MPNSTs, including P53 (*TP53*), epidermal growth factor receptor (*EGFR*), cell surface glycoprotein (*CD44*), hepatocyte growth factor (*HGF*), cyclin-dependent kinase inhibitor 2a (*CDKN2A*), C-X-C chemokine receptor 4 (*CXCR4*), MET proto-oncogene (*MET*), platelet-derived growth factor receptors alpha and beta (*PDGFRA* and *PDGFRB*), RB transcriptional corepressor 1 (*RB1*), and SRY-box transcription factor 9 (*SOX9*) [14,15,16,17,18]. In addition, numerous comparative studies between benign neurofibromas and MPNSTs identified the differential expression of genes, including KIT proto-oncogene (*KIT*), ERB-B2 receptor tyrosine kinase 2 (*ERBB2*), met proto-oncogene (*MET*), transforming growth factor beta 1 (*TGFB1*), hepatocyte growth factor (*HGF*), tenascin XB (*TNXB*), TSC complex subunit 2 (*TSC2*), tenascin C (*TNC*), mechanistic target of rapamycin kinase (*MTOR*), phosphatase and tensin homolog (*PTEN*), forkhead box M1 (*FOXM1*), BCL2 like 1 (*BCL2L1*), PDZ-binding kinase (*PBK*), cyclin-dependent kinase 4 (*CDK4*), BUB1 mitotic checkpoint serine/threonine kinase B (*BUB1B*), and NIMA-related kinase 2 (*NEK2*) [19,20,21,22,23,24,25,26,27,28]. However, the other key factors closely involved in the progression of benign PNs to MPNSTs remain unclear.

In our previous study, based on reverse transcription–polymerase chain reaction (RT-PCR)-based differential display analysis, we found that interferon-induced transmembrane protein 1 (IFITM1) was downregulated in MPNST tissues of patients with NF1 compared to that in benign tumor tissues [29]. Although several studies have suggested that interferon-inducible IFITM1 may play a role in antiproliferation and in cell adhesion signals [30,31,32], the involvement of IFITM1 in MPNST pathogenesis remains unexplored. In this study, we investigated the hypothesis that the reduction of IFITM1 in MPNSTs is associated with the activation of the Ras/ERK signaling pathway, which is known to be involved in the malignant transformation of NF1. First, we assessed the basal expression levels of IFITM1 in the PN and MPNST tissues of six patients with NF1 and NF1-associated Schwann cell lines. Next, we examined whether IFITM1 levels were inversely correlated with Ras/ERK signaling activation by manipulating IFTM1 expression in normal and NF1-associated MPNST cells. Finally, we demonstrated that interferon-gamma (IFN-γ), which is known to induce IFITM1 expression, suppressed tumor progression in xenograft mice injected with MPNST cells.

## 2. Results

### 2.1. Downregulation of IFITM1 in MPNST Tissues of Patients with NF1

Basal expression of IFITM1 was examined in tumor tissues obtained via surgical resection of six patients with NF1 at Ajou University Hospital. The clinical characteristics of the patients are summarized in Table 1. All patients were diagnosed with NF1 via assessment of representative NF1 clinical features, including café-au-lait spots, neurofibromas, and *NF1* gene mutations. Histopathologically benign and malignant tumor tissues were confirmed via hematoxylin and eosin (H&E) staining and IHC with the Schwann cell marker S100 (Table 1). 

**Figure 1:**
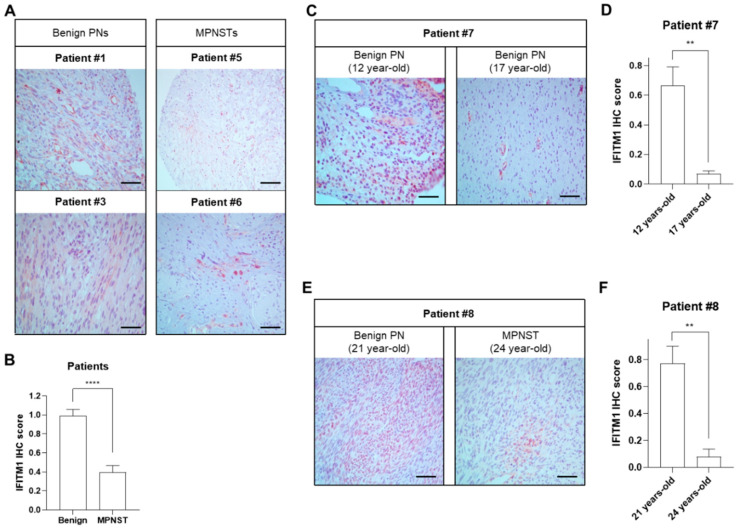
Comparison of IFITM1 protein levels between tissues of benign plexiform neurofibromas (PNs) and malignant peripheral nerve sheath tumors (MPNSTs) from patients with NF1. (**A**,**C**,**E**) Immunohistochemical staining images of IFITM1 in the tumor tissue specimens of patients. Scale bar = 60 μm (400×). (**B**,**D**,**F**) Quantitative analysis of immunohistochemistry results of IFITM1 staining. IFITM1-stained positive cells (brown) and nuclei (blue) were analyzed in four different parts of the slides and the immunohistochemical score of IFITM1 was calculated using ImageJ software (Version 1.53t). The data are presented as the mean ± standard error of mean (SEM). Two-tailed paired *t*-test was used for statistical analysis. ** *p* < 0.01 and **** *p* < 0.0001.

IFITM1 protein levels were significantly decreased in MPNST tissues (P5 and P6) compared to those in benign PN tumor tissues (P1 and P3) (Figure 1A,B). To investigate the hypothesis that the expression levels of IFITM1 may change according to tumor progression, IFITM1 protein levels were compared in two tumor tissues obtained at two different time points with a 5-year interval from the same patient. In patient P7, the expression levels of IFITM1 were significantly lower in the late stage than in the early stage, although both tissues were benign PNs (Figure 1C,D). Similarly, in patient P8, IFITM1 expression was much lower in the MPNST tissue at 24 years of age than that in the benign PN tissue at 2 years of age. These results indicate that IFITM1 was downregulated in MPNSTs compared to that in PNs, suggesting that IFITM expression may be involved in tumor progression in NF1.

### 2.2. Close Association between IFITM1 Protein Levels and Ras/ERK Signaling in Normal and MPNST Schwann Cell Lines

**Figure 2:**
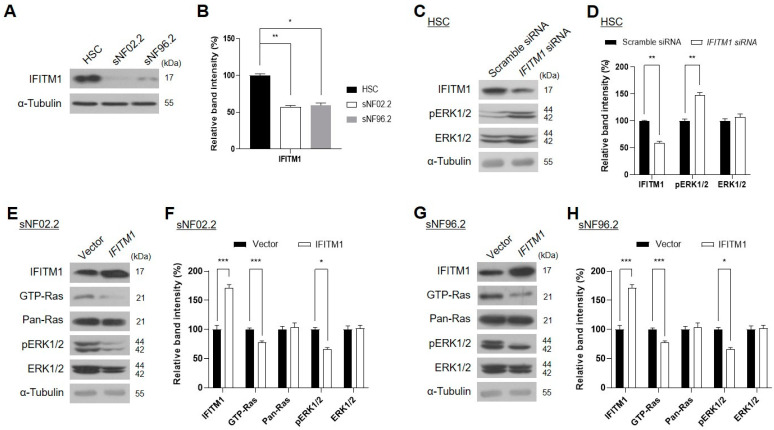
Association of IFITM1 protein levels with Ras signaling protein levels and activation in the normal human Schwann cell (HSC) and NF1-associated malignant peripheral nerve sheath tumor (MPNST) Schwann cell lines. (**A**,**C**,**E**,**G**) Western blot analysis results of IFITM1 and Ras signaling-associated proteins in normal HSC and NF1-associated MPNST cells (sNF02.2 and sNF96.2). α-tubulin was used as the internal control. The molecular weight of the proteins is expressed as *kilodalton* (kDa). pERK—phosphorylated ERK. (**A**) Basal protein levels of endogenous IFITM1 were assessed in normal and MPNST Schwann cells. (**C**) Normal HSC cells were transfected with nonspecific scrambled siRNA (50 nM) or *IFITM1* siRNA (50 nM). After 72 h of incubation, the protein levels were assessed via Western blotting. (**E**,**G**) Two NF1-associated MPNST sNF02.2 and sNF96.2 cells were transiently transfected with an empty vector or *IFITM1* plasmid. After 24 h of incubation, the transfected cells were lysed, and the protein levels were assessed via Western blotting. (**B**,**D**,**F**,**H**) Immunoblot band density analysis results of the tested proteins. Relative band intensity was quantified using ImageJ software after normalization with the intensity of α-tubulin. One-way analysis of variance followed by Tukey’s honest significant difference post-hoc test was used for statistical analysis in (**B**) and two-tailed paired *t*-test was used for statistical analyses in (**D**,**F**,**H**). * *p* < 0.05, ** *p* < 0.01, and *** *p* < 0.001.

Further, we examined the downregulation of IFITM1 in MPNST Schwann cell lines. The basal protein levels of IFITM1 were assessed in normal and MPNST Schwann cells. Endogenous IFITM1 levels were markedly lower in the two MPNST Schwann cell lines (sNF02.2 and sNF96.2) than those in the normal human Schwann cells (HSCs) (Figure 2A,B), demonstrating that IFITM1 was downregulated in both NF1-associated MPNST tissues and cells. 

Many studies have reported that the overactivation of Ras/ERK signaling causes tumorigenesis and malignant progression in many tumors [33,34,35,36,37,38]. Thus, the accumulation of GTP-Ras, the active form of Ras, may be an indicator of tumor progression [39]. We investigated whether IFITM1 expression affects Ras and its downstream ERK1/2 protein levels and activation. As GTP-Ras is undetectable in normal HSCs, the phosphorylation levels of ERK1/2 were evaluated instead. The knockdown of *IFITM1* via siRNA treatment of normal HSCs resulted in an increase in phosphorylated ERK1/2 (Figure 2C,D). Conversely, *IFITM1* overexpression in the two *MPNST* Schwann cell lines resulted in a decrease in GTP-Ras and phosphorylated ERK1/2 in both sNF02.2 (Figure 2E,F) and sNF96.2 (Figure 2G,H) cells. These results suggest the close involvement of IFITM1 in the regulation of the Ras/ERK signaling pathway, which is involved in NF1 pathogenesis.

### 2.3. *IFN*-*γ* Upregulates IFITM1 and Inactivates Ras/ERK Signaling in MPNST Schwann Cells

**Figure 3:**
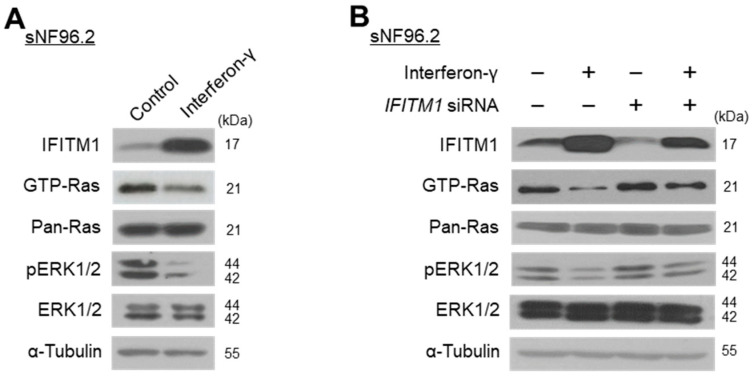
Effect of interferon-gamma (IFN-γ) treatment on IFITM1 expression and Ras signaling-associated protein levels and activation in malignant peripheral nerve sheath tumor (MPNST) cells. (**A**) NF1-associated MPNST NF96.2 cells were treated with IFN-γ (1000 U/mL) for 3 h and protein levels were assessed via Western blotting. (**B**) sNF96.2 cells were transfected with *IFITM1* siRNA (50 nM) for 72 h with (+) or without (−) additional treatment with IFN-γ (1000 U/mL) for 3 h. The negative symbol (−) for IFN-γ indicates a distilled water treatment, while that for *IFITM1* siRNA indicates a scrambled siRNA treatment (50 nM). The levels of IFITM1, GTP-Ras, Pan-Ras, phosphorylated ERK1/2 (pERK1/2), ERK1/2, and α-tubulin were assessed via Western blotting.

Previous studies reported that treatment with interferon induces IFITM1 expression [31,40] and suppresses the MEK/ERK pathway [41,42]. We investigated whether IFN-γ could result in IFITM1 upregulation and Ras/ERK signaling inactivation in NF1-associated MPNST cells. Treatment of sNF96.2 cells with IFN-γ for 3 h resulted in markedly increased IFITM1 and decreased GTP-Ras and phosphorylated ERK1/2 levels (Figure 3A). Furthermore, in *IFITM1* knockdown cells, IFN-γ treatment resulted in increased IFITM1 expression and decreased Ras and ERK1/2 activation (Figure 3B). These results indicate that IFN-γ treatment caused IFITM1 upregulation and Ras/ERK signaling inactivation. 

### 2.4. *IFN*-*γ* Suppresses Tumor Progression in Xenograft Mice Injected with NF1-Assocaited MPNST Cells

To investigate the effects of IFN-γ on IFITM1 expression in MPNST progression in vivo, we generated xenograft nude mice using the NF1-associated MPNST S462 cell line. In a previous study, the S462 cell line was characterized as a cancer stem cell-like cell with the potential to differentiate into S100-positive Schwann cells [43]. Treatment of S462 cells with IFN-γ resulted in increased IFITM1 levels and decreased Ras activation and phosphorylated ERK1/2 levels (Supplementary Figure S1).

**Figure 4:**
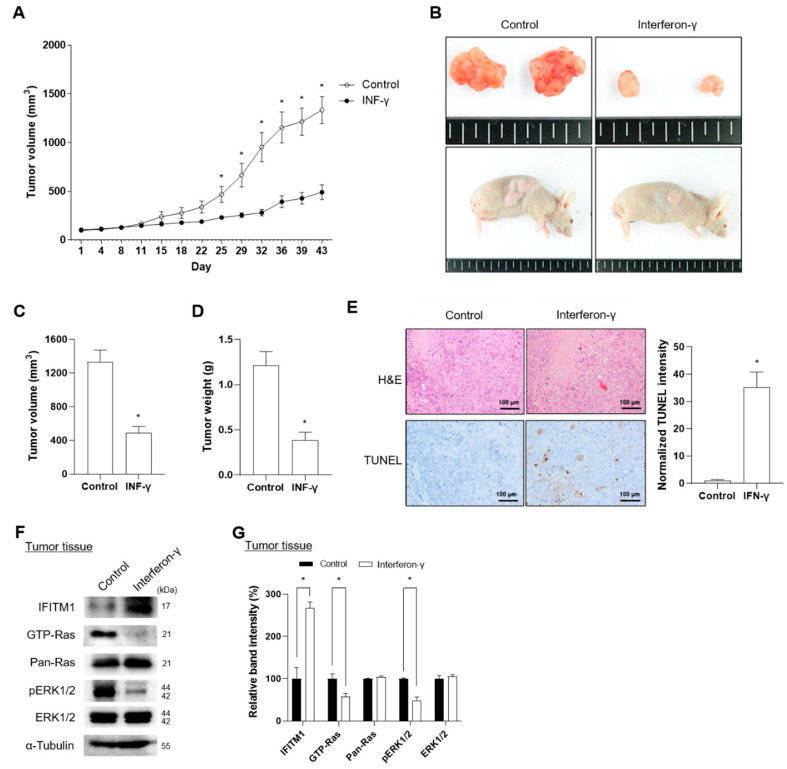
Effect of interferon-gamma (IFN-γ) on IFITM1 expression and tumor progression in xenograft mice injected with MPNST S462 cells. (**A**) Xenotransplantation of 5-week-old BALB/c nude mice was conducted by injecting S462 cells into the right flank. The tumor volume of the xenograft mice was measured every 3 d for 43 d. For the experimental mice group, IFN-γ (33,000 IU/kg) was administered thrice a week for 4 weeks. (**B**–**D**) On the final day of the experiment, the mice were sacrificed, and tumor tissues were dissected. The shapes of mice and dissected tumors were photographed, and tumor volume and weight were measured. (**E**) Histological changes were evaluated via H&E staining for tumor necrosis and by TUNEL staining assay for tumor cell apoptosis. Scale bar = 100 μm. Positive cells for TUNEL (brown) and nuclei (blue) staining were analyzed on three independent slides and the normalized intensity of TUNEL staining was quantified using ImageJ software. (**F**) Changes in IFITM1, Ras, and ERK1/2 protein levels were analyzed in the dissected tumor tissues. (**G**) Relative band intensity was quantified using ImageJ software after normalization with the intensity of α-tubulin. Two-tailed paired *t*-test between control and IFN-γ treated mice was used for statistical analysis. * *p* < 0.05.

Xenograft mice were successfully generated via injection of S462 cells and were confirmed based on tumor shape, volume, and weight (Figure 4A–D). IFN-γ was subcutaneously injected thrice a week for four weeks. The body weight did not differ between the nontreated control and IFN-γ-treated groups during the observation period, including receipt, final quarantine, transplantation, and group assignment day (Supplementary Figure S2). Notably, IFN-γ treatment successfully suppressed tumor volume during days 25 to 43 of transplantation (Figure 4A). At the end of IFN-γ treatment, tumor tissues were dissected, and tumor volume and weight were measured. After 43 d of treatment, IFN-γ-treated tumor tissues presented a 2.7-fold decrease in tumor volume and a 3.1-fold reduction in tumor weight compared with nontreated control tissues (Figure 4B–D). Tumor progression status was histologically evaluated via H&E staining for necrosis of tumor cells and TUNEL assay for apoptosis of tumor cells. IFN-γ treatment showed significantly increased TUNEL-positive cells in tumor tissues; however, in H&E staining, no difference was found in single and contiguous cells between the control and IFN-γ-treated groups, indicating that IFN-γ induced apoptosis rather than necrosis (Figure 4E). Consistent with in vitro results, IFN-γ treatment increased IFITM1 expression and decreased GTP-Ras and phosphorylated ERK1/2 (Figure 4F,G). 

Collectively, these results suggest that IFN-γ treatment suppresses tumor progression through induction of IFITM1 expression and subsequent inhibition of Ras and ERK1/2 activation.

## 3. Discussion

Previous studies reported that IFITM1 expression is responsive to IFNs α and γ and that downregulation of IFITM1 was detected in cervical cancer and hepatocellular carcinoma [31,32]. IFN-γ induces a decrease in the intracellular calcium pump and consequently causes antiproliferative effects in cytokine-induced human salivary gland cells [44]. Elevated IFITM1 levels were associated with improved survival outcomes in patients with chronic myeloid leukemia and glioblastoma [30,45]. In NF1 studies, several data showed that IFN-γ treatment or IFN-γ gene transfection directly inhibited the proliferation of NF1-associated MPNSTs or neurofibroma cells [46,47,48]. 

However, the molecular function of IFN-γ-mediated IFITM1 at the cellular level during tumor progression remains controversial. Several studies have shown the involvement of IFITM1 in the progression of colorectal cancer and resected gastric and esophageal adenocarcinomas [49,50,51]. These reports suggested that IFN-γ-responsive genes may have important roles in the malignant progression of tumors. These contrasting results may be explained by cell type specificity. Studies showing the positive role of IFITM1 in tumor progression have mainly used epithelial cells and fibroblasts. However, NF1 studies, including the current study, mainly used Schwann cells because they are considered the main cell type of tumor origin [52]. Therefore, we focused on all experiments involving Schwann cells in tumor tissues and cell lines. IFITM1 expression in tumor tissues was examined in S100-positive Schwann cells. 

In this study, we found that IFITM1 was downregulated in MPNST tumor tissue samples from patients with NF1 and NF1-associated MPNST Schwann cell lines (Figure 1 and Figure 2). Overexpression of IFITM1 in *NF1*-associated MPNST cells caused a significant decrease in Ras activation (GTP-Ras) and downstream ERK1/2 phosphorylation, indicating that the expression level of IFITM1 was inversely correlated with tumor progression in NF1 (Figure 2). IFN-γ treatment significantly induced IFITM1 expression in MPNST cells and xenograft mice, and this IFN-γ-mediated IFITM1 upregulation may have caused the suppression of Ras and ERK1/2 (Figure 3 and Figure 4). 

MPNST is associated with a poor prognosis and is the leading cause of mortality in patients with NF1 [53]. Owing to their aggressive and invasive nature and tendency for local relapse, the treatment of NF1-associated MPNSTs, which include surgery, radiotherapy, immunotherapy, and chemotherapy, lacks data regarding improved overall survival in patients [54]. Chemotherapy alone or in combination with anticancer drugs, such as doxorubicin, carboplatin, dactinomycin, ifosfamide, gemcitabine, ocetaxel, etoposide, cisplatin, vincristine, cyclophosphamide, imidazole, and carboxamide, has been used; however, data on their efficacy are limited [54]. As targeted therapeutic strategies, clinical trials with MEK inhibitors, such as sorafenib and selumetinib; the mTOR inhibitor sirolimus, the VEGF-A inhibitor bevacizumab; the Hsp90 inhibitor ganetespib; the selective c-KIT inhibitor pexidartinib; and the multi-kinase inhibitor pazopanib have been explored; however, effective response data are not available yet [54].

Clinical trials of polyethylene glycolylated interferon-alpha-2b have been conducted in patients with NF1-related PN [55]. However, clinical trials of IFN-γ for NF1 treatment have not been conducted. IFN-γ plays a key role in the activation of cellular immunity and stimulation of antitumor immune response [56]. IFN-γ has been used in clinical trials because it contributes to the efficiency of cancer immunotherapy and enhances tumor senescence and apoptosis of cancer cells and inhibits metastasis and angiogenesis [56]. Recently, results of a phase 1 study of the combination of IFN-γ and nivolumab for patients with advanced solid tumors have reported that the therapy was safe and revealed a modest benefit and that IFN-γ induction increased circulating chemokines [57]. Based on our in vivo results in xenograft mice, IFN-γ, alone or in combination with other anticancer drugs, may be a promising clinical trial candidate for the treatment of NF1 patients with MPNSTs. 

Collectively, our results suggest that IFITM1 acts as a negative regulator of Ras/ERK signaling in MPNST cells and xenograft mice. Therefore, IFITM1 may be a potential biomarker for evaluating tumor progression in NF1. More importantly, IFN-γ, which can effectively induce IFITM1 expression, may be a good candidate for the therapeutic treatment of MPNSTs in NF1. 

## 4. Materials and Methods

### 4.1. Patient Samples

This study was approved by the Institutional Review Board of Ajou University Hospital (AJIRB-GEN-GEN-11-321 and AJIRB-GEN-SMP-11-107), and informed consent was obtained from all patients. Initially, tumor tissue samples from eight patients (P1–P8) diagnosed with NF1 in the Department of Pathology at Ajou University Hospital were obtained through surgical resection. However, the tissue samples from patients P2 and P4 were not suitable for study because of their low quality and were omitted. Therefore, only the samples from the other six patients with NF1 (P1, P3, P5, P6, P7, and P8) were finally studied.

### 4.2. Immunohistochemistry (IHC)

The benign PN tumor and MPNST tissue samples obtained from the six patients diagnosed with NF1 were fixed with 10% formalin (Thermo Fisher Scientific, Kalamazoo, MI, USA) and embedded in paraffin (Sigma-Aldrich, St. Louis, MO, USA) blocks. Three-micrometer sections were prepared and stained with H&E (Thermo Fisher Scientific, Kalamazoo, MI, USA). For immunohistochemical analysis, paraffin blocks were deparaffinized and rehydrated. Antigen retrieval was performed by boiling the slides. Immunostaining was performed using the UltraVision LP-HRP polymer 3,3-diaminobenzidine (DAB) kit (Thermo Fisher Scientific, Kalamazoo, MI, USA). Paraffin samples were incubated with Ultra V Block (Thermo Fisher Scientific, Kalamazoo, MI, USA) for 5 min to block nonspecific reactions at room temperature. The samples were incubated with the primary antibody for 1 h, followed by incubation with HRP polymer-conjugated secondary antibody for 30 min. The slides were imaged using DAB, and nuclear counterstaining was performed using hematoxylin. The cells were counted using ImageJ software (Version 1.53t)(National Institutes of Health, Bethesda, MD, USA), and the IFITM1 IHC score was obtained by dividing the number of IFITM1 (brown; diaminobenzidine)-positive cells by that of nuclear (blue)-positive total cells.

### 4.3. Reagents and Cell Lines

IFN-γ human recombinant protein (RIFNG100) was purchased from Thermo Fisher Scientific, (Kalamazoo, MI, USA), and NF1-MPNST sNF96.2 and S462 cells were treated with 1000 U/mL of IFN-γ for 3 h.

HSCs were obtained from ScienCell Research Laboratories and cultured in Schwann cell medium with 1% Schwann cell growth supplement. NF1-associated MPNST sNF96.2 and sNF02.2 cell lines were purchased from American Type Culture Collection (Manassas, MD, USA) and maintained in Dulbecco’s modified eagle medium (DMEM) (Thermo Fisher Scientific, Kalamazoo, MI, USA) with 10% fetal bovine serum (Thermo Fisher Scientific, Kalamazoo, MI, USA). The cancer stem-cell like NF1-associated MPNST S462 cell line was gifted by Dr. Lan Kluwe (University Medical Center, Hamburg-Eppendorf, Hamburg, Germany) and was cultured in DMEM with 10% fetal bovine serum. 

### 4.4. Quantitative Reverse Transcription-Polymerase Chain Reaction (qRT-PCR)

Total RNA was extracted using the TRIzol reagent (15596026; Invitrogen, Carlsbad, CA, USA). The extracted RNA (2 µg) was treated with DNase I (Thermo Fisher Scientific, (Kalamazoo, MI, USA) and reverse transcribed by using the iScript™ cDNA Synthesis Kit (K1632; Fermentas, Burlington, ON, Canada). qRT-PCR was performed using SYBR Premix Ex Taq (#RR420A; Takara, Otsu, Japan) on a 7500 Fast Real-Time PCR System (Applied Biosystems, Foster City, CA, USA). The gene-specific primers used were as follows: 5′-GGAGCGAGATCCCTCCAAAAT-3′ and 5′-GGCTGTTGTCATACTTCTCATGG-3′ for *GAPDH* and 5′- CCAAGGTCCACCGTGATTAAC-3′ and 5′-ACCAGTTCAAGAAGAGGGTGTT-3′ for *IFITM1*. 

### 4.5. Short Interfering RNAs (siRNAs), Plasmids, and Transfection

The siRNAs were obtained from Bioneer Inc. (Seoul, Republic of Korea). The siRNA sequences were as follows: 5′-CCTACGCCACCAATTTCGT-3′ for the nonspecific scrambled control siRNA and 5′-AACTCATGACCATTGGATTCA-3′ for the *IFITM1* siRNA. Human *IFITM1* cDNA (NCBI Gene ID: 8519) was amplified via RT-PCR using the primers 5′-TTCTCGAGCTATGCACAAGGAGGAACATGAGGTGG-3′ and 5′-CTGGATCCAGCATTGCACAGTGGAGTGCA-3′. The PCR product was digested with restriction enzymes XhoI and BamHI and inserted into a pcDNA3.1(-) vector (Clontech Laboratories, Mountain View, CA, USA). 

HSCs were transfected with nonspecific scrambled control siRNA (50 nM) or *IFITM1* siRNA (50 nM) for 72 h using Lipofectamine RNAiMAX (13778-075; Invitrogen, Carlsbad, CA, USA). NF1-associated MPNST cells were transfected with an empty pcDNA3.1 vector or *IFITM1* plasmid for 24 h using Lipofectamine 2000 (11668-019; Invitrogen, Carlsbad, CA, USA). The transfected Schwann cells were harvested and prepared for subsequent analyses.

### 4.6. Ras Activation Assay and Western Blot Analysis

The levels of activated GTP-Ras were analyzed using a Ras activation assay kit (#17–218; Upstate Biotechnology, Lake Placid, NY, USA) according to the manufacturer’s protocol. The harvested cells were lysed in lysis buffer (provided) on ice. The extracted protein lysates (400 μg) were isolated for immunoprecipitation of GTP-Ras and were incubated with Raf-1-RBD agarose beads (provided) for 1 h at 4 °C. The beads were rinsed thrice with lysis buffer (provided) and resuspended in 2 × Laemmli sample buffer. The prepared proteins were analyzed using western blotting.

Cells and tumor xenograft tissues were harvested and lysed in radioimmunoprecipitation assay buffer containing a protease inhibitor cocktail (11836170001; Roche Applied Science, Penzberg, Germany) and 1 mM phenylmethylsulfonyl fluoride (10837091001; Roche Applied Science, Penzberg, Germany). Total extracted proteins were quantified using Bradford assay (#5000001; Bio-Rad Laboratories, Hercules, CA, USA). The extracted proteins samples (20–40 μg) were loaded onto 10–12% polyacrylamide gels, run on sodium dodecyl sulfate–polyacrylamide gel electrophoresis, and electroblotted onto polyvinylidene fluoride (PVDF) membranes (Millipore, Billerica, MA, USA). The electroblotted PVDF membranes were blocked with 5% bovine serum albumin (Sigma-Aldrich, St. Louis, MO, USA) for 1 h. The prepared membranes were then incubated for 24 h in a cold room with a primary antibody: IFITM1 (Boster Biological Technology, Pleasanton, CA, USA); extracellular signal-regulated kinase 1/2 (ERK1/2) (#9102; Cell Signaling Technology, MA, USA); phosphorylated ERK1/2 (#4376; Cell Signaling Technology); S100 (#MA5-12969; Thermo Fisher Scientific, Kalamazoo, MI, USA); Pan-Ras (#17-218; Upstate Biotechnology, Lake Placid, NY, USA); α-tubulin (sc-5286; Santa Cruz Biotechnology, Santa Cruz, CA, USA), HRP-conjugated goat anti-rabbit IgG (sc-2004; Santa Cruz Biotechnology, Santa Cruz, CA, USA); and HRP-conjugated goat anti-mouse IgG (sc-2005; Santa Cruz Biotechnology, Santa Cruz, CA, USA). The membranes were visualized using enhanced chemiluminescence (ECL) Western Blotting Detection reagents (16028; Intron Biotechnology, Gyeonggi-do, Republic of Korea), and the relative band intensity (%) of specific proteins was quantified after normalization using ImageJ software (National Institutes of Health, Bethesda, MD, USA).

### 4.7. Animal Study

Animal experiments were approved by the Institutional Animal Care and Use Committee (IACUC) of Biotoxtech Inc. (Cheongju, Republic of Korea) and processed by Biotoxtech Inc. under committee guidelines (Approval No.: 170552). Five-week-old specific pathogen-free BALB/c nude mice were maintained in a filter-top cage under controlled conditions (12 h light/12 h dark cycle) and provided with 18% protein rodent diet (Teklad, Madison, WI, USA) and sterilized water ad libitum. S462 cells (1 × 10^6^ cells) were injected into the right flank of the mice, and the tumor volume was measured using a caliper (Mitutoyo, Kawasaki, Japan) using the following formula: tumor volume (mm^3^) = length (mm) × width^2^ (mm^2^) × 0.5. When the average tumor volume was between 80–120 mm^3^, the mice were randomized and divided into control and IFN-γ treated groups (n = 5 in each group). Mice were treated with IFN-γ (33,000 IU/kg) via subcutaneous injection thrice a week for 43 d, and all mice were sacrificed at the end of the experimental period for histological analysis. Tumor tissues from the xenograft mice were dissected, harvested, and fixed with 10% formaldehyde for 24 h. Formalin-fixed paraffin embedded (FFPE) tissues (4 μm) were stained with H&E. To evaluate apoptosis in tumor xenograft tissues, FFPE tissues were subjected to a TUNEL assay (ab206386; Abcam, Cambridge, UK), according to the manufacturer’s instructions. TUNEL-positive cells were visualized under a light microscope (Leica Microsystems, Wetzlar, Germany). Tumor necrosis was examined based on morphological changes, such as nuclear collapse or loss and cytoplasmic eosinophilic changes.

### 4.8. Statistical Analysis

The results are presented as the mean ± standard error of the mean (SEM). Statistical differences were assessed using SPSS 11.0 for Windows (IBM Corp., Armonk, NY, USA). Multiple groups were compared using one-way analysis of variance (ANOVA), followed by Tukey’s honest significant difference post-hoc test, with a provability value (*p* value) < 0.05 was considered significant.

## 5. Conclusions

IFITM1 is significantly downregulated in MPNST tissues from patients with NF1 and in NF1-associated MPNST cell lines. Treatment of MPNST cell lines and MPNST cell-injected xenograft mice with IFN-γ resulted in increased IFITM1 expression and decreased Ras and ERK1/2 activation. IFN-γ successfully inhibited tumor progression in the xenograft mice. These results suggest that IFITM1 is closely involved in MPNST pathogenesis and that IFN-γ can effectively suppress the progression of benign PN tumor to MPNST in NF1 through IFITM1 induction and subsequent negative regulation of the Ras/ERK signaling pathway. 

## Figures and Tables

**Table 1:** Histological, clinical, and genotypic characteristics of the six patients with NF1.

Patients			Histological Findings			Clinical Features							Genotype
ID	Gender	Age at Diagnosis	H&E	S100	IFITM1	Café-Au-Lait Spots	Neuro-Fibromas	Freckling	Optic Glioma	Lisch Nodule	Skeletal Dysplasia	Family History	*NF1* Gene Mutation
P1	Male	59	Benign	+	++	Y	Y	Y	N	N	N	Y	N/A
P3	Female	5	Benign	+	++	Y	Y	Y	N	N	N	Y	N/A
P5	Male	32	Malignant	+	+	Y	Y	N	N	N	N	N	c.4861_4862GT>AG
P6	Female	41	Malignant	+	+	Y	Y	Y	N	N	N	N	N/A
P7	Male	12	Benign	+	++	Y	Y	Y	N	Y	Y	N	c.4537C>T
		17	Benign	+	+	Y	Y	Y	N	Y	Y	N	c.4537C>T
P8	Male	21	Benign	+	++	Y	Y	Y	N	Y	N	N	c.6792C>A
		24	Malignant	+	+	Y	Y	Y	N	Y	N	N	c.6792C>A

Abbreviations: NF1—neurofibromatosis type1; H&E—hematoxylin and eosin; N/A—not analyzed; +—expression positive; ++—relatively higher expression; Y—patient with the indicated clinical features; N—patient without the indicated clinical features.

## Data Availability

The data presented in this study are available upon request from the corresponding author.

## Supplementary Materials

The following supporting information can be downloaded at: https://www.mdpi.com/article/10.3390/ijms25179265/s1.
