# Supplementary material for: Interferon-Induced Transmembrane Protein 1 (IFITM1) Is Downregulated in Neurofibromatosis Type 1-Associated Malignant Peripheral Nerve Sheath Tumors
Source: Int J Mol Sci. 2024 Aug 27;25(17):9265. doi: 10.3390/ijms25179265 (PMC11395022; doi:10.3390/ijms25179265)
Supplement: Supplementary file 1 [file ijms-25-09265-s001.zip › ijms-3149047-supplementary.pdf]

Supplementary Figures

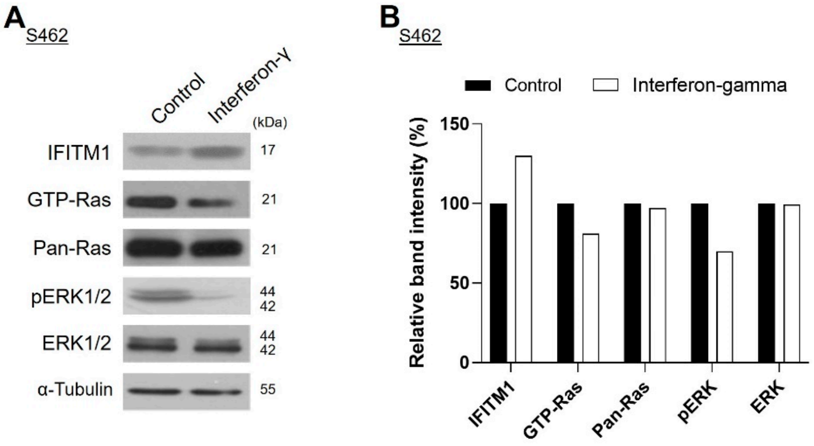

Figure S1. Effect of interferon-gamma (IFN-γ) treatment on IFITM1 expression and Ras signaling-associated protein levels and activation in the NF1-associated S462 MPNST cell line. (A) S462 cells were treated with IFN-γ (1,000 U/mL) for 3 h and the levels of IFITM1, GTP-Ras, Pan-Ras, phosphorylated ERK1/2(pERK1/2), ERK1/2, and α-tubulin were assessed via western blotting. (B) Immunoblot band density of the tested proteins was normalized to the intensity of α-tubulin (internal control) for each sample. The relative band intensity was quantified using ImageJ software.

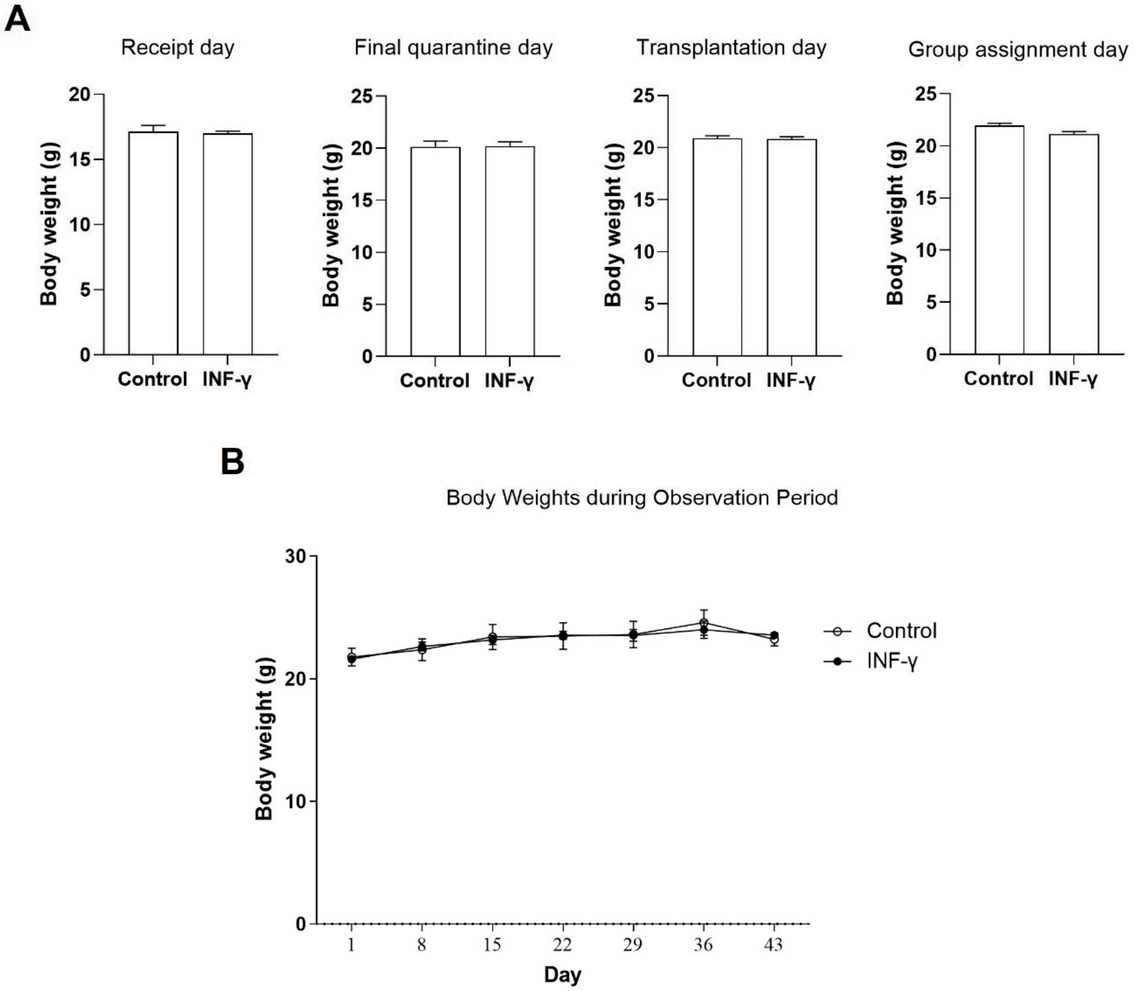

Figure S2. Effect of interferon-gamma (IFN-γ) treatment on body weight in NF1-associated MPNST xenograft mice. (A) Body weight did not differ between the nontreated control and IFN-γ-treated groups, including the days of receipt, final quarantine, transplantation, and group assignment. (B) Body weight during the entire observation period.
